# Low-cost and prototype-friendly method for biocompatible encapsulation of implantable electronics with epoxy overmolding, hermetic feedthroughs and P3HT coating

**DOI:** 10.1038/s41598-023-28699-6

**Published:** 2023-01-30

**Authors:** Marek Novák, Jozef Rosina, Hana Bendová, Kristina Kejlová, Alena Vlková, Marian Rucki, Lada Svobodová, Robert Gürlich, Jan Hajer

**Affiliations:** 1 Department of Biomedical Technology, Faculty of Biomedical Engineering, Czech Technical University in Prague, Kladno, Czech Republic; 2 Department of Medical Biophysics and Medical Informatics, Third Faculty of Medicine, Charles University, Prague, Czech Republic; 3 Department of Health Care and Population Protection, Faculty of Biomedical Engineering, Czech Technical University in Prague, Kladno, Czech Republic; 4 Centre of Toxicology and Health Safety, National Institute of Public Health, Prague, Czech Republic; 5 Department of General Surgery, Third Faculty of Medicine, Charles University, University Hospital Královské Vinohrady, Prague, Czech Republic; 6 Department of Internal Medicine, Third Faculty of Medicine, Charles University, University Hospital Královské Vinohrady, Prague, Czech Republic

**Keywords:** Biomedical engineering, Electrical and electronic engineering, Mechanical engineering, Implants

## Abstract

The research of novel implantable medical devices is one of the most attractive, yet complex areas in the biomedical field. The design and development of sufficiently small devices working in an in vivo environment is challenging but successful encapsulation of such devices is even more so. Industry-standard methods using glass and titanium are too expensive and tedious, and epoxy or silicone encapsulation is prone to water ingress with cable feedthroughs being the most frequent point of failure. This paper describes a universal and straightforward method for reliable encapsulation of circuit boards that achieves ISO10993 compliance. A two-part PVDF mold was machined using a conventional 3-axis machining center. Then, the circuit board with a hermetic feedthrough was placed in the mold and epoxy resin was injected into the mold under pressure to fill the cavity. Finally, the biocompatibility was further enhanced with an inert P3HT polymer coating which can be easily formulated into an ink. The biocompatibility of the encapsulants was assessed according to ISO10993. The endurance of the presented solution compared to silicone potting and epoxy potting was assessed by submersion in phosphate-buffered saline solution at 37 °C. The proposed method showed superior results to PDMS and simple epoxy potting.

## Introduction

In recent years, a distinct portion of research in the biomedical field pursued the development and prototyping of implantable medical devices, primarily biosensors and actuators (i.e. neurostimulators). In commercialized implantable devices, the most common materials used for encapsulation are glass and titanium due to their extremely low permeability for water and biocompatibility^1^. However, the fabrication of these hermetic enclosures is tedious and expensive^2^, especially for small prototype runs intended for research purposes.

However, having a hermetic (or close to hermetic) and biocompatible material that encapsulates the device solves only half of the problem. With a few exceptions, all implantable medical devices require cable feedthroughs to expose sensing elements to the surrounding tissue or to deliver electrical stimulation signals. The most considerable issue is the isolation of electronics from the external environment. Simple designs which encapsulate the device with cables without any protection against liquid ingress are prone to liquid exposure of the electronics. This can be limited by careful design choices and FEM simulations which can detect failure modes, such as fractures and delamination^3^.

A review article by. Ahn et al.^2^ provides an overview about various processes and materials that are used or could be used for manufacturing of medical devices. The inorganic materials (such as Al2O3, SiO2 relies mostly on ALD—“atomic layer deposition” and/or CVD—“chemical vapor deposition”—which are expensive and not widely available, especially for small-scale prototyping or even one-off prototypes. Long-term tests indicated that thin PDMS coating^4^ does not provide sufficient protection against moisture without the so-called PDMS caulking that was performed by Parylene CVD coating. Other polymers that were tested for biocompatible encapsulation include polyimide (requiring 400 °C temperature to achieve a successful bond which limits the applications to bare integrated circuits) and LCP (liquid crystal polymer) encapsulation that requires thermal bonding including application of pressure. PDMS is usually applied in two ways—spin coating for thin layers and potting for thick layers.

A lot of current research in implantable devices resorts to simple PDMS potting, direct spraying, or brush coating^5–7^ of fully assembled devices. While this is the easiest method which also provides a soft, non-traumatizing surface of the implant, it has an inherent disadvantage of very high permeability to water, around two orders of magnitude higher than epoxies have^8^. Also, creating sealed feedthroughs in silicone is very challenging because of very low surface energy and thus, adhesion to other materials^9^. Mechanical stressing of the flexible feedthrough may also lead to premature failure, especially when using polymers that might exhibit low surface energy. Special adhesives or primers must be usually used when a failure-proof bond to glass/metal/polymer is desired. However, this introduces chemicals that might harm the biocompatibility of the device. The combination of high water vapor permeability and low adhesion to substrates leads to the formation of air bubbles on the surface of the encapsulated electronics where water can condense and subsequently cause premature failure^7,10^. In digital electronic circuits, the inherent immunity of digital communication buses to leakage current may allow operation with some liquid present on the board even though it will shorten the lifetime of the device due to corrosion. However, sensitive analog circuits with high impedance inputs can be severely affected by a small leakage current. Any leakage currents also directly translate to higher power consumption and thus, lower battery life. Failure due to corrosion of the printed circuit board or components is also common^7^. Some research also uses glass vial packaging with subsequent epoxy potting of the opening^11^ which limits the shape of the device.

There is also prior research that describes successful lab fabrication of a hermetically sealed implantable device, but often commenting the encapsulation as very tedious, especially when creating hermetic wire feedthroughs^9,12^. Using PDMS for encapsulation may force researchers to provide excessively thick encapsulant layers and wire feedthrough lengths (at least a few millimeters) to achieve sufficient protection. An easier and prototype-friendly method for reliable small-scale manufacturing which would allow encapsulant thicknesses of 0.5 mm or less could foster further development in the field, allowing to create smaller implants or use the extra space to increase battery capacity and/or to add functionality. Simple potting using a Petri dish or a simple one-part mold has been also used^13^ and successfully validated in terms of functionality but such molding technique creates a sharp edge at the top of the device and also forces the design to have a flat top which is undesirable for some applications. A smooth and curved design of the encapsulated device without any traumatizing (i.e. sharp) edges can substantially reduce foreign body rejection by the soft tissue^2,14^. Some sources mention hand coating of the epoxy on the surface of the electronic package without any control over the final shape of the device and control over the design of feedthroughs^15^. A use of a PTFE mold for epoxy potting was reported for neurostimulation implants but the shape of the molded device was limited to a ring with sharp edges^16^.

In the presented method which is both prototype-friendly and can be used for semi-production runs, the printed circuit board is encapsulated in an ISO10993-compliant epoxy with the help of a small CNC-machined injection mold from polyvinylidene fluoride (PVDF). The use of an injection mold enables full freedom of shape, unlike methods which rely on coating or simple potting only. The presence of sharp corners due to electronic components may increase rejection rate of the implantable device. In the presented method, the biocompatibility can be then further enhanced by providing a thin layer of biocompatible P3HT—poly(3-hexylthiophene-2,5-diyl)—polymer which is formulated in an ink and dip-coated on the surface of the epoxy. This material was used because of ability to tailor its cell-surface adhesion properties with further surface modification^17^. The direct capillary path for liquid ingress to the electronics is severed with a miniature glass-Kovar compression hermetic feedthrough which is directly molded into the encapsulated device. Sometimes, the biocompatibility of the implantable device is assumed based on the used materials and its certifications. This assumption may lead to issues as the biocompatibility can be affected by any process or contact with a contaminant, starting with improper mixing of the multi-part epoxy/PDMS, wrong curing scheme leading to a high concentration of unreacted monomers/oligomers, machining of the mold with contaminated tools and/or coolant, using improper coolant, curing inhibition caused by incompatibility between epoxy/PDMS and the encapsulated device etc. A lot of current research does not evaluate the biocompatibility of the final product and relies only on known biocompatibility of the base material. To thoroughly evaluate the potential of the presented method, a full description of the presented method with subsequent qualification of finalized devices according to ISO10993 was performed to prove its feasibility with an emphasis on short to medium term in vivo experiments (the potential water ingress was assessed for 15 days).

This method is intended for researchers who develop custom implantable medical devices or are looking for a biocompatible method of encapsulation of sensors, connector assemblies, etc. The method is demonstrated on a mock-up of an implantable medical device consisting of two simple circuits that are used to characterize the durability of the encapsulation. Other researchers replicating this method are encouraged to modify the design of tooling and the used materials according to their specific needs.

## Materials and methods

All experimental procedures involving animals were conducted under standard environmental conditions in the accredited Animal Facility of the NIPH, Prague, Czech Republic (16OZ23091/2017-17214) in compliance with the European rules of animal care and welfare (Directive 2010/63/EU). The studies were authorized by the Ministry of Health of the Czech Republic (LLNA-MZDR 6593/2019-4/OVZ, irritation test by intracutaneous (intradermal) administration-MZDR 60413/2017-3/OVZ). After experiments, the test animals were separately sedated by isoflurane (5%) and subsequently humanely killed with an overdose of CO_2_. The experimental procedures involving animals followed the ARRIVE guidelines.

The selection of volunteers and the test methods complied with the WMA Declaration of Helsinki (1964, amended 2013) and the International Ethical Guidelines for Health-related Research Involving Humans (CIOMS, 2016). The study was performed in accordance with ISO 14155 (2020) and was approved by the Ethical Review Committee of the National Institute of Public Health. The 30 volunteers gave their written informed consent before their participation in the study was permitted.

### Construction of the printed circuit boards

The printed circuit boards (PCBs) implement a simple RC circuit with a diode. A composite drawing of the schematic and circuit diagram are available as a Supplementary Figure S1. The purpose of the circuit board is to provide a test platform to easily detect any ingress of moisture that would change the electrical behavior or corrode the components and copper traces when exposed to saline solution. Also, no solder mask or tin/electroless gold plating was done to intentionally increase the vulnerability of the traces to corrosion. Normally, a solder mask and electroless nickel gold (ENIG) coating of all exposed conductors is advisable due to its electrochemical neutrality.

**Figure 1:**
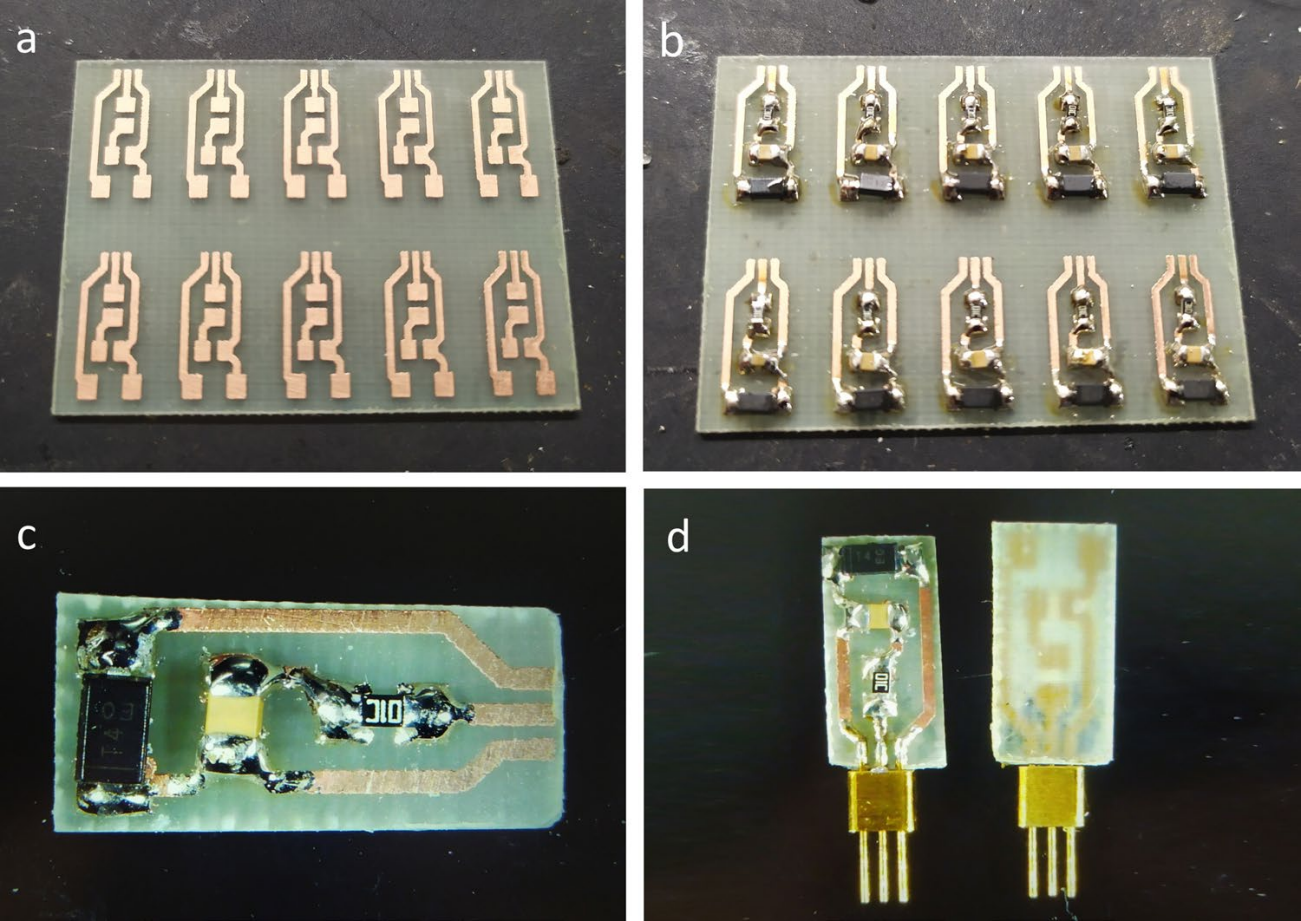
Fabrication of the printed circuit boards; (**a**) unpopulated board; (**b**) populated board; (**c**) detail of a depanelized board; (**d**) hermetic feedthrough soldered to the PCB.

A total of 12 depanelized PCBs were manufactured which were divided into four groups—silicone (PDMS) encapsulation (“PDMS1-3”), epoxy-only encapsulation (“EPOXY1-3”), epoxy encapsulation with hermetic feedthroughs (“HERM1-3”), and control—no encapsulation and exposure to the saline solution (“CTRL1-3”). The hermetic feedthroughs were obtained from Xi’an Elite Electronics, type JMC-1553. The process of PCB population, depaneling, and soldering of the hermetic feedthrough for the HERM group is shown in Fig. 1. In other groups, 34AWG FEP-coated cable was soldered.

After soldering, all PCBs were cleaned in an isopropyl alcohol bath at 60 °C for 15 min, then in Shesto UTFLU ultrasonic cleaning bath (5% solution in DI water) for 10 min at 50 °C and 50 W ultrasound power. The use of a specific product for flux removal is mandatory because pure alcohol does not fully remove flux and other contaminants. After that, residual water and flux remover were expelled by soaking the devices in fresh room-temperature isopropyl alcohol for 15 min under magnetic stirring. Then, the PCBs were depanelized with scissors, the edges were lightly sanded to remove any burrs, and washed again in isopropyl alcohol. After that, the PCBs were stored in a dust-free environment. Gloves must be used throughout the cleaning process and when handling the PCBs to avoid contamination which might alter the electrical properties that will be measured later and to avoid deposits which might compromise the epoxy bond.

### Construction of the mold and encapsulated devices manufacturing

Next, a PVDF mold was machined on a 3-axis CNC router with a machining vise and 3-jaw lathe chuck. A PVDF rod with a diameter of 60 mm was used to machine both halves of the mold. PVDF was chosen due to its extremely low coefficient of friction and surface energy which enables easy de-molding without silicone sprays or similar de-molding agents. If a standard oil or synthetic-based water emulsion flood coolant is not available or might be contaminated from previous machining, a high-pressure air mist with a 50:50 isopropyl alcohol/DI water mixture can be used to cool the tool and reduce cutting forces. As this coolant mixture does not have rust-inhibiting properties, it is important to proceed with the machining as quickly as possible and after finishing the work, to wipe off any water residue from the machine, including wet PVDF chips. Also, it is required to treat any steel parts with a penetrating oil spray (such as WD-40) or flood coolant to prevent damage to the machine. In the case of low-cost CNC routers built from aluminum extrusions, less care can be taken as aluminum parts will not corrode. If a standard coolant of MQL (minimum quantity lubrication) is used, extra care should be practiced during the cleaning of the finished mold to avoid contamination. Also, if the machine was previously used for manufacturing of metal parts, small metal fragments can be present in the coolant that might embed into the softer plastic.

**Figure 2:**
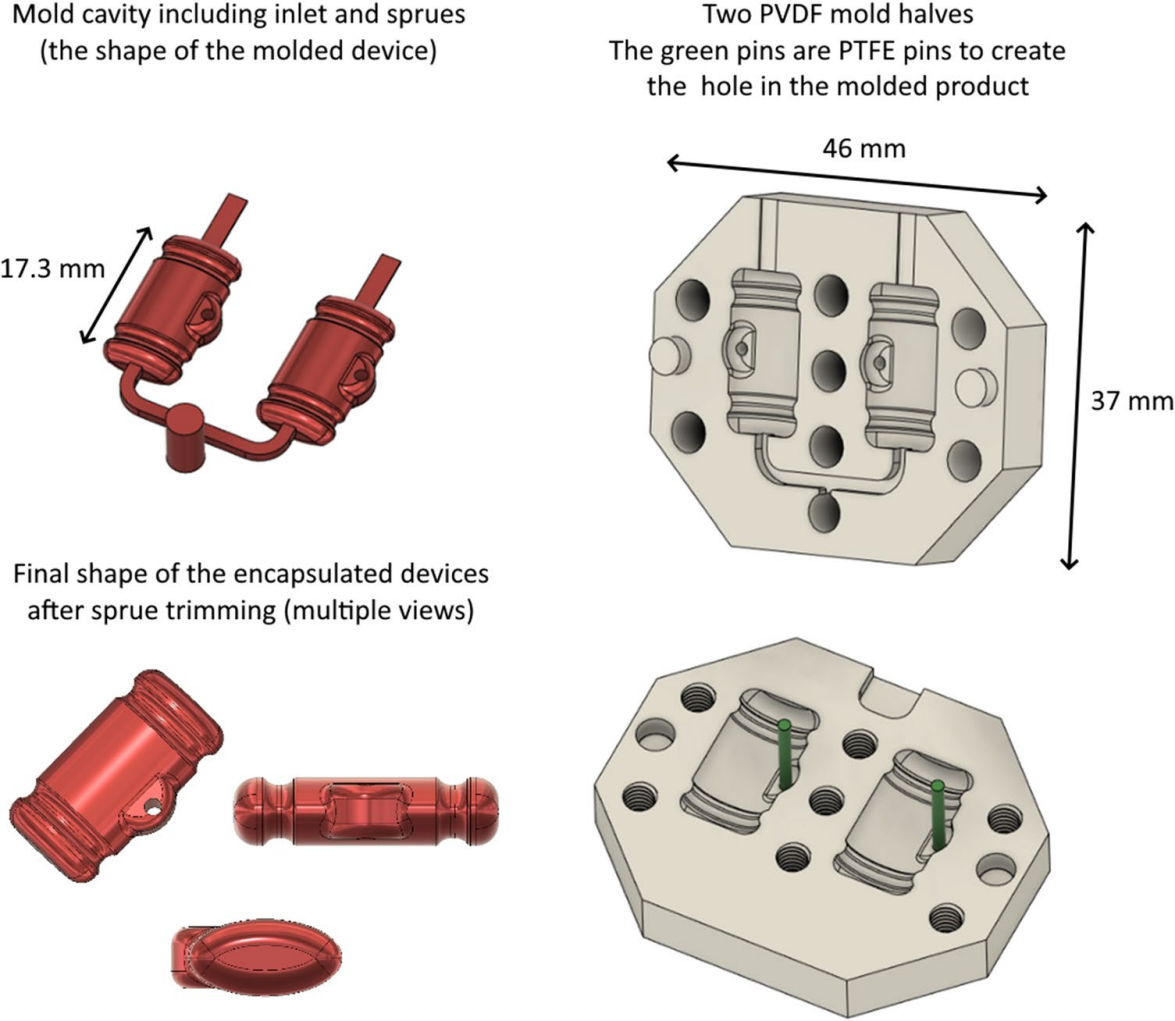
Injection mold design.

**Figure 3:**
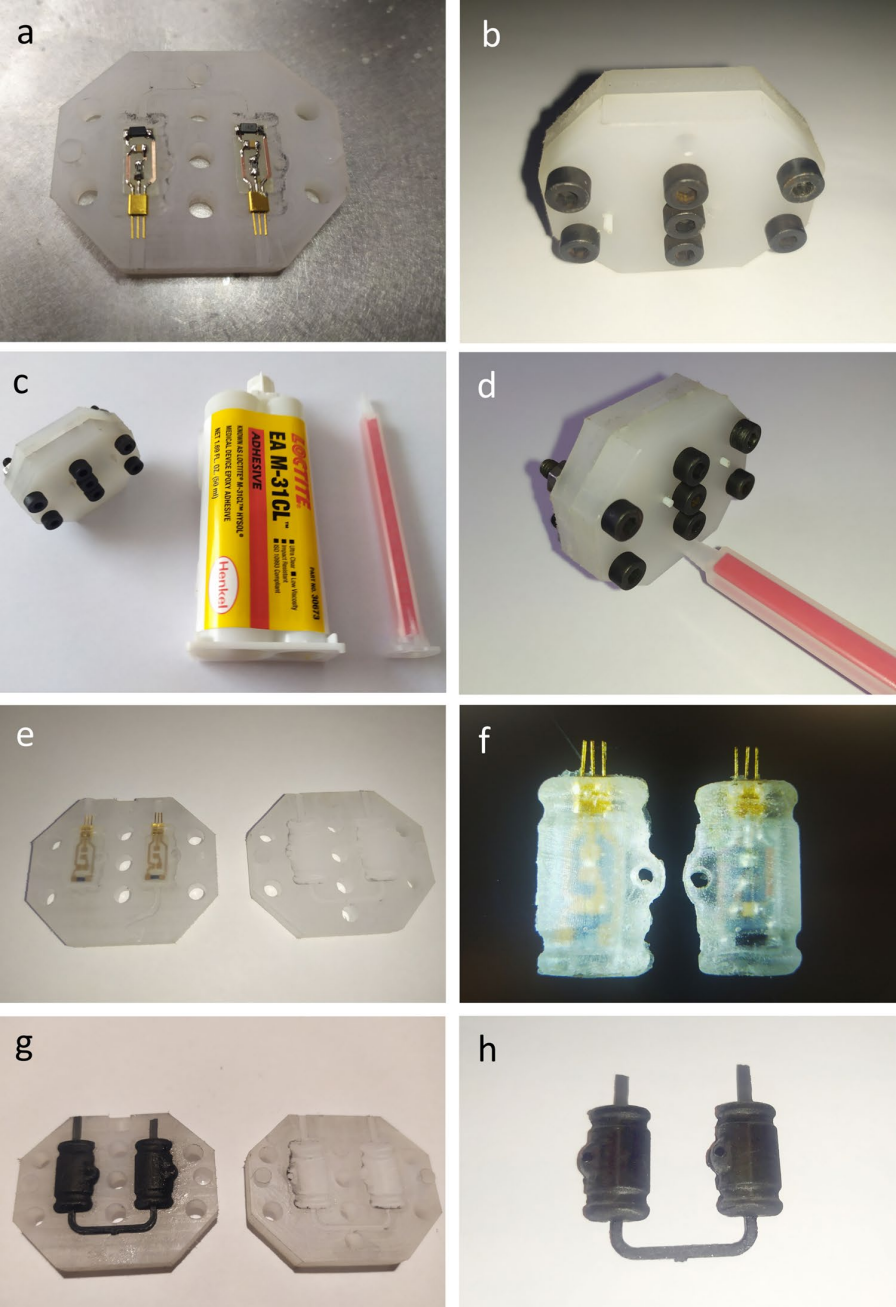
Encapsulation process; (**a**) PCBs inserted to the mold; (**b**) mold closed with side pins present; (**c**) epoxy cartridge with the mold; (**d**) insertion of the static mixer to the gate; (**e**) after mold halves separation; (**f**) detailed view of encapsulated devices; (**g**) test run with black PDMS to provide better visibility; (**h**) PDMS parts with visible sprues and part of the gate.

The design of the tool for injection molding is shown in Fig. 2. The 3D model in STEP format is provided as Supplementary Information S2. The beige color indicates two PVDF mold halves, the green color indicates two PTFE side pins for the formation of a side hole in the finished part and the red color shows the expected final shape of the encapsulated product, including gate and sprues. Two halves enclose the two cavities which are filled with the encapsulant. Like during the design of a regular plastic injection mold, a side gate design with two sprues and gates in the bottom middle part was used. Due to the low rigidity of PVDF, both halves are held together by 8 bolts to reduce or avoid the formation of “flashes” (formation of a very thin edge of material between the mold halves). An alternative way to press the two halves of the mold together is to not use bolts but to compress the two halves together in a vise. The inlet for the epoxy was designed to match the mixers which are supplied with the epoxy cartridges, as shown in Fig. 3d.

A total of 5 tools were used for machining of the mold. Only tools with diameter less than 6 mm were used to show the viability of the method with economical aluminum extrusion based 3-axis CNC routers (commonly known in the industry by generic names “CNC3020”, “CNC4030” etc.). The toolpaths were generated by the CAM environment of the Autodesk Fusion 360 software. For facing, boring of holes and general roughing, a 3-flute 4 mm diameter HSS end mill and 3 mm diameter end mill were used. To machine the sprues, outlet channels where the hermetic feedthrough or cable is placed and to machine the holes for side pins, a 4 flute 1 mm diameter HSS end mill was used. To machine the inner shape of the cavity, a 4 flute 1.5 mm diameter HSS ball mill was used. The threads were done manually with a hand spiral tap. The side PTFE pins were machined using the 4 mm end mill with an outside boring operation. The specific cutting parameters (rotational speed of the tool, feed rates, chip per tooth rate, width of cut, etc.) should be determined based on the tool manufacturer’s recommendation and experimenting. Lower rigidity machines may generally require lower feed rates and higher rotation speeds of the tools. However, too high rotational speeds may lead to melting of the material due to excessive heat. Exercise caution when using power tools and wear recommended personal protective equipment (i.e. eye protection).

The mold is designed to accommodate two populated PCBs. The design of the final encapsulated devices was chosen arbitrarily to show various features which can be achieved with this technology. The top half of the mold contains the inlet which is a hole of 4 mm in diameter. A static epoxy mixer is inserted into the inlet before injection. The flow of epoxy is then divided into two (or more in the case of multi-cavity molds) sprues (channels) that transport the epoxy to the part entry (“gate”). The gate opens to the mold cavity where the injected epoxy creates the desired part shape. The general rule of thumb from our findings is that the sprue and gate cross-section should be between 1 and 3 mm^2^. Smaller cross-section leads to higher injection pressures while larger ones increase epoxy losses. The top half also contains two locating pins and 8 holes for M4 bolts. The bottom half of the mold is considerably simpler. It does contain two locating holes that fit into the pins in the other half of the mold. Instead of M4 bolt holes, threads are provided. The small recess on the top is used to insert a tool to split the two halves easily during demolding. Two holes, one for each finished part, are drilled throughout the mold. To this hole, a small PTFE pin is placed. This creates a side hole in the finished part which could be used for a thread or to assist otherwise during immobilization of the implantable device. The two tapered parts on both sides can be used for endoscopic graspers or snares. The cable or hermetic feedthrough was used to hold the PCB in the middle of the mold, so the epoxy encapsulates the electronics completely.

After placing two populated PCBs in the mold and closing of the mold with bolts, PDMS (Easycomposites AS40) or epoxy (Loctite EA M-31 CL) was injected through the inlet port until it started pouring out of the top area, indicating a complete fill. For epoxy, a static mixer was used which mitigated formation of bubbles. For PDMS, the two components were first mixed in a separate container, vacuumed for a few minutes to deareate the PDMS and then transferred to the syringe, preferably from the bottom of the container (where the concentration of air bubbles should be lowest). The epoxy was chosen based on its biocompatibility and advised use in assembly of medical devices. The PDMS was chosen due to very low shrinkage, being and addition cure PDMS. Addition cured PDMS that uses platinum-based catalyst does not require water/moisture presence to cure or acetoxy curing that leaves corrosive acetic acid in the vicinity of the curing silicone. The unreacted mixture should be injected slowly to avoid the formation of air bubbles which would make a complete fill impossible. In case of the presented mold, the advised injection time from start to finish was around 30 s. Then, the epoxy resin cartridge was retrieved together with the applicator and the inlet was covered with a piece of tape to avoid leakage of the epoxy. Curing can be done either at room temperature for 24 h or at an elevated temperature of 60 °C for 4 h.

**Figure 4:**
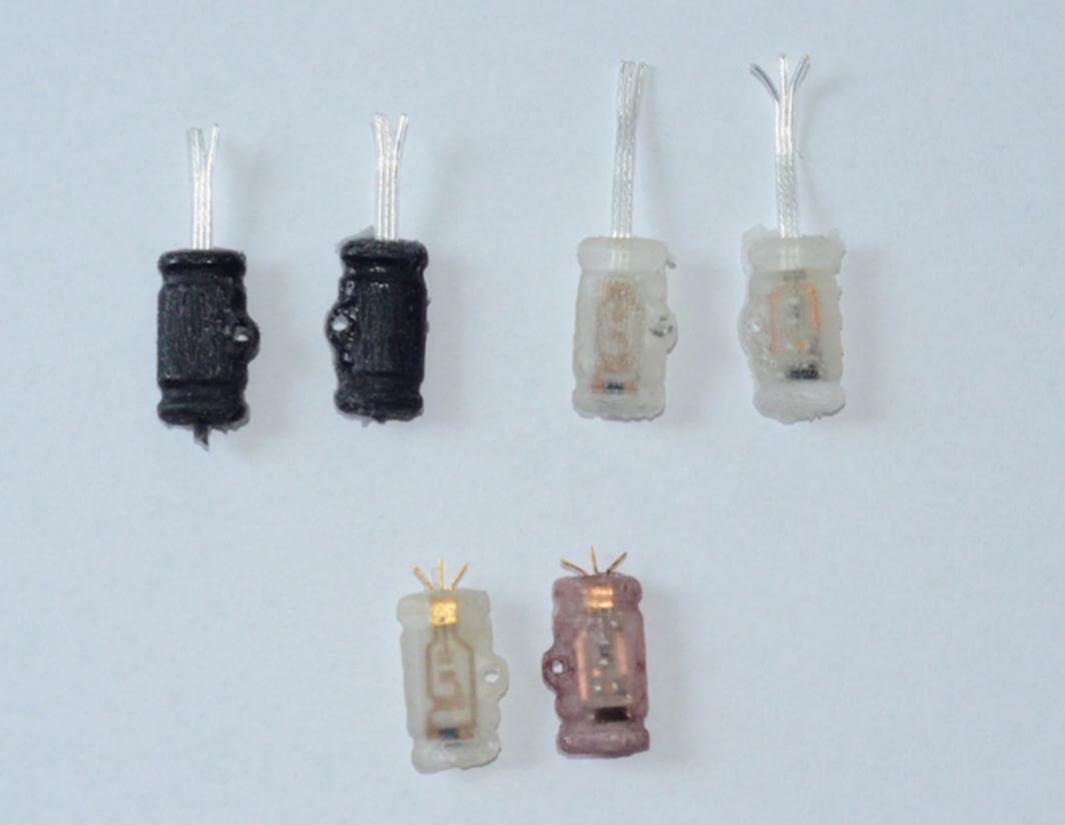
Various encapsulated devices—PDMS, epoxy and epoxy with hermetic feedthroughs and optional P3HT coating.

After curing, first, all bolts were removed from the mold. Then, a flat screwdriver was inserted in the slot in the top part of the mold and the two halves were slowly expanded to split the two halves of the mold. Based on the initial experiments, it is advised to apply small force over a longer period rather than excessive force quickly to avoid mechanical damage to the mold and/or the encapsulated devices. The whole molding and demolding process is visualized in Fig. 3. All types of encapsulated devices that were subjected to performance testing are shown in Fig. 4. When the top part of the mold was released, the inlet and sprues were dislodged before dislodging the encapsulated devices. Then, the gates were trimmed with a sharp scalpel and the edges were sanded if excessive material was still present. On the opposite end of the encapsulated devices, the process was similar. Preliminary testing of the circuit was done at this point to verify its functionality. Any possible unreacted monomers and oligomers were washed by rinsing the encapsulated devices in isopropyl alcohol and water several times. The mold was then mechanically cleaned to remove any left bits of the epoxy and subsequently washed thoroughly with isopropyl alcohol and water to remove any residues. The mold should not be stored with tightened bolts to avoid permanent stress to the material which could cause preliminary failure of the threads in the threaded half of the mold.

### P3HT coating formulation and application

The encapsulated devices can be further coated with P3HT (poly(3-hexylthiophene-2,5-diyl, 94.2% regioregular from Ossila), a polymer known to be biocompatible^18^. To avoid expensive and often sparsely available vacuum processes (i.e. CVD of PVD) which are commonly used for deposition of thin films of Parylene (another biocompatible polymer used for coating production-grade medical devices)^19^, a method that uses an ink formulated from a P3HT polymer which can be then applied to the treated surface was used.

First, 0.05 g of the P3HT polymer was dissolved in 24.95 g of chloroform at room temperature with a magnetic stirrer. The exact ratio is not critical as partial evaporation of the solvent is unavoidable. A watch glass placed on the beaker was used to limit the evaporation of the solvent. After 5 min of stirring, the P3HT ink solution was placed in an airtight glass container and stored under refrigeration. Caution during handling must be exercised as the ink is volatile, flammable, and toxic due to presence of chloroform. Working in a fume hood with suitable personal protective equipment is required.

The coating of the encapsulated circuit boards was performed by dipping the encapsulated devices in the solution for three seconds. Then, the device was retrieved and held still until the solvent evaporated from the surface. During evaporation, the color changed from dark orange (dissolved state of the polymer) to deep purple color (dried state). Subsequent post-drying in a convection oven at 50 °C for 30 min was performed to remove the residual solvent. After that, the polymer can be cleaned off the gold-plated hermetic feedthroughs with fresh chloroform. This procedure can also be used for any part of the encapsulated device (external electrodes, sensors, etc.).

### Biocompatibility testing

The biocompatibility tests were performed at the National Institute of Public Health, Laboratories of Toxicology, Šrobárova 49/48, 100 00 Prague 10, Czech Republic, Laboratory No.1206, accredited by Czech Accreditation Institute according to EN ISO/IEC 17025:2017. The biocompatibility of the materials was assessed using standard 25 × 25 × 1 mm square samples manufactured with an identical technique. A total of 5 molds were made from the PVDF rod with the use of the same tooling, coolant, and machine. The epoxy was then poured into the molds and left to cure at room temperature. A total of 200 square samples were made. These were divided into two groups—“implant-epoxy” and “implant-p3ht”. The first group contained pure epoxy samples while the latter group was coated in P3HT according to the procedure described in “P3HT coating formulation and application”.

#### Cytotoxicity

Cytotoxicity in vitro was assessed in accordance with ISO 10993-5^20^. Briefly, after 24 h cultivation (37 °C, 7.5% CO_2_) the 3T3 Balb/c cell culture (recommended for the test by Annex A of the standard ISO 10993-5) was exposed to the test material extracts (extracted according to ISO 10993-12 in the ratio of 3 cm^2^/ml DMEM with serum for 24 h at 37 °C) and controls (sodium lauryl sulfate as positive control, DMEM with serum as negative control)^21^. At the end of the 24 h treatment the cells were stained by Neutral Red dye according to DB-ALM Protocol No. 46 (0.2 ml Neutral Red solution per well, 3 h incubation, Neutral Red desorb solution–ethanol/acetic acid). The degree of cytotoxicity (i.e. the decrease of cell viability) was quantitatively determined as the Neutral Red uptake measured by fluorescence-luminescence reader FLX800TBI (BioTek). The viability of cells exposed to the test material extracts reaching 70% or more in comparison to the negative control (DMEM with serum) confirms the absence of cytotoxicity.

#### Skin irritation in vivo

Irritation test by intracutaneous (intradermal) administration on animals was performed in accordance with ISO 10993-23^22^. The test materials were extracted according to ISO 10993-12^23^ in the ratio of 6 cm^2^/ml (exaggerated ratio mimicking the worst case scenario) using polar solvent (saline solution) and non-polar solvent (cottonseed oil) for 72 h at 37 °C. Extracts and solvents were injected intracutaneously on the back of 3 animals (albino rabbits, and females, after 5 days of acclimatization) at five sites in the amount of 0.2 ml, i.e. twenty application sites on each animal. The appearance of each injection site was observed at 24 ± 2 h, 48 ± 2 h, and 72 ± 2 h after injection, and tissue reactions were graded according to the "Grading system for intracutaneous (intradermal) reactions". Individual mean scores for the test sample and solvent were calculated. The final test sample score was calculated as the difference between the test sample mean score and solvent (blank) mean score. If the final test sample score is 1.0 or less, the sample is considered as non-irritant.

Evaluation of human skin irritation was performed according to ISO 10993-23 in a group of 30 healthy volunteers. The selection of volunteers and the test methods complied with the WMA Declaration of Helsinki (1964, amended 2013)^24^ and the International Ethical Guidelines for Health-related Research Involving Humans (CIOMS, 2016)^25^. The study was performed in accordance with ISO 14155 (2021)^26^ and was approved by the Ethical Review Committee of the National Institute of Public Health. The volunteers gave their written informed consent before their participation in the study was permitted. The materials (2.5 × 2.5 cm) and positive control (20% SDS, 0.4 ml per patch) were applied to the upper outer arm using Hill Top Chambers (containing a gauze pad, diameter 1.8 cm). The patches were applied progressively starting with a duration of 15 min and 30 min, up to 1 h, 2 h, 3 h, and 4 h. The skin irritation potential hazard was determined by comparison of the number of volunteers that produced skin reaction (e.g. erythema, edema, dryness) after the test material application and the number of volunteers that produced skin reaction after the positive control application at intervals of 24 ± 2 h, 48 ± 2 h and 72 ± 2 h after patch removal. Fisher´s exact test was used for the statistical evaluation of the results.

#### Skin sensitization in chemico—DPRA

The DPRA was performed according to OECD TG 442C^27^ with minor modifications. The assay was performed in cysteine-only modification to predict the potential of in chemico interactions, comparing the values of tested samples with the measured activity of the negative control (acetonitrile). Peptides containing cysteine were prepared and purified by GenScript (Piscataway, NJ). Briefly, 50 µl of the tested extract (extracted according to ISO 10993-12 in the ratio of 3 cm^2^/ml in saline solution for 72 h at 37 °C) was mixed with 750 µl of peptide solution (0.5 mM in sodium phosphate buffer, pH 7.5) and 200 µl of acetonitrile and incubated in the dark for 24 h at 25 °C. Following incubation, the relative peptide concentration was measured by reverse-phase high-performance liquid chromatography (HPLC, Ecom HPLC) on a Chromolith-C18 column (5.0 mm × 100 mm) with gradient elution and UV detection at 220 nm (Ecom UV/VIS detector) using an external standard linear calibration curve. Three samples were prepared from each tested extract and each sample was measured in triplicate. Cysteine peptide percent depletion values were then calculated and used in a prediction model to categorize a substance into one of four reactivity classes (< 13.89%, minimal,13.89–23.09%, low; 23.09–98.24%, moderate; > 98.24%, high reactivity class), which distinguish sensitizers and non-sensitizers (OECD, 2019).

#### Skin sensitization in vitro—LuSens

The LuSens assay^28,29^ was performed according to OECD TG 442D^30^. The cells were kindly provided by BASF SE (Germany) and tested for mycoplasma contamination during routine use. Briefly, after 24 h cultivation (37 °C with 5% of CO_2_), the keratinocyte cell line LuSens was exposed to the test material extracts (extracted according to ISO 10993-12 in the ratio of 3 cm^2^/ml in DMEM and DMSO for 24 h at 37 °C). DMSO extracts were diluted in D-MEM before adding to the plate, resulting in a final non-cytotoxic DMSO concentration of 1%. After 48 h the luciferase activity was measured using One-Glo luciferase substrate (Promega) by a plate reader (GLOMAX Multi Reader, Promega). In parallel, the cell viability was determined by MTT assay, the resulting formazan concentrations were measured by Eon High Performance Microplate Spectrophotometer (BioTek Instruments) at 570 nm. A positive control, ethylene glycol dimethacrylate inducing luciferase expression above 2.5-fold in comparison to the vehicle controls, and negative control, dl-lactic acid (5000 μM), were included in each test run. Two independent experimental runs were carried out with each sample tested in triplicate. For the acceptance of the assay, at least 3 tested concentrations with viability above or equal to 70% must be available. The sensitizing potential of the tested extract is indicated if the luciferase activity equals or exceeds a 1.5-fold induction compared to the appropriate vehicle control at concentrations that do not reduce cell viability under 70%29, (Urbisch et al.^29^, Ramirez et al.^31^).

#### Skin sensitization in vivo—LLNA:DA

The LLNA: DA in vivo test, based on the determination of the number of proliferating cells in the lymph nodes by measuring intracellular ATP (adenosine triphosphate) using a bioluminescence method, was performed according to ISO 10993-10^32^ and OECD TG 442A^33^. Healthy female Balb/c mice (Charles River Laboratories, Germany), aged 8–12 weeks, acclimatized for 7 days, were used in the experiment. Four animals were used per each experimental group (negative control—saline solution/oil, positive control—dinitrochlorobenzene, test groups—treated with the extract in polar extractant (saline solution) and in non-polar extractant (cottonseed oil), extracted according to ISO 10993-12 in the ratio of 3 cm^2^/ml solvent for 72 h at 37 °C.

Briefly, all the test samples were applied to the dorsal part of both ears of animals in each test group in the amount of 25 µl per ear on Day 1, 2, 3, and 7 of the experiment. On Day 8, the test animals were separately sedated by isoflurane (5%) and subsequently humanely killed with an overdose of CO_2_, the auricular lymph nodes were removed and a cell suspension was prepared, diluted, transferred into three parallel wells of a white microtiter plate (100 µl per well), and mixed with 100 µl of Cell Titer-Glo Luminescent Reagent (PROMEGA). Bioluminescence (in relative luminescence units) was measured by a luminometer (GloMax-Multi Detection System, Promega). The results were expressed as Stimulation Index (SI), which was obtained by comparing the average values of the test group or the positive control group with the negative control group.

### Performance testing

To assess the performance of different encapsulation materials and technologies, a similar methodology that was used in prior research^5^ was designed and used. All implantable devices were incubated at 37 °C while immersed horizontally (to promote water ingress) in phosphate-buffered saline solution at a pH of 7.4 which corresponds to the normal pH of blood. At several distinct data points (before immersion, after 10 min, 1 h, 4 h, 1 day, 5 days, and 15 days), the electrical parameters of the embedded electronic circuit were assessed by electrical measurement. Before the measurement, exposed terminals or exposed ends of the cable were briefly sprayed with DI water and dried with a paper towel. The measurement accuracy of the used meter (Lutron LCR-9184) at the selected measurement frequency of 100 Hz and range of up to 200 nF and 20 kOhm is ± (0.5% + 5 digits). The RMS voltage of the excitation waveform is 600 mV. For capacitance, these uncertainty values are valid when the dissipation factor is less or equal to 0.1. In this scenario, the dissipation factor is going to be around 0.63 (100 nF capacitor and 10 kOhm resistor in series at 100 Hz,the ESR of a ceramic capacitor at this frequency is negligible). Thus, based on the manufacturer’s recommendations, it was necessary to compensate for this by multiplying by a factor $\sqrt{1+{D}^{2}}=1.18\to \mathrm{approx}.\pm \left(0.6\mathrm{\% }+ 5\mathrm{ digits}\right)$. The final accuracy of the meter for the selected measurement range was then $\pm \left(0.6\mathrm{\% }+ 0.05\mathrm{ nF}\right)$ for capacitance and $\pm \left(0.5\mathrm{\% }+ 0.05\mathrm{ kOhm}\right)$ for resistance.

The performance of the diode circuit was measured by measuring the forward voltage at 1 mA current (to determine if the diode circuit is still functional) and after that, a short reverse DC pulse of 30 V with measurement of current after 100 ms to determine the presence of the leakage current caused by liquid ingress. The measurement was performed with a Keysight DSOX1102 oscilloscope. The current was determined by measuring voltage across 10 kOhm resistor placed in series with the circuit. This method of measurement introduces an error due at higher currents due to substantial voltage loss across the 10 kOhm shunt resistor. However, the measurement only indicates a presence or non-presence of current. For this reason, this measurement method was deemed as acceptable for the purpose of this study. The current of 1 mA is the industry standard for measurement of forward diode voltage and the 30 V reverse voltage was chosen with respect to the expected maximum voltage that will be present in implantable devices. A literature review in this area suggested that in most implantable devices, maximum voltage is limited to several volts and some stimulators use voltages up to 30 V to provide sufficient current when delivering neurostimulation pulses to high impedance tissue^34–36^. The dwell time between application of the voltage and measurement of 100 ms was chosen to measure the current under stable conditions, without presence of transient effects caused by linear lab power supply power-up. Next, to assess possible change in dielectric breakdown strength of the epoxy, the diode circuit was tested at the maximum permissible voltage of the used 1N4148 diode (100 V). The two other terminals were connected to maximize the E-field present at the hermetic feedthrough (0.3 mm which equals to 333 V/mm E-field).

Prior to the experimenting, the acceptance criteria (when the encapsulation was considered as not failed) for the measurement of ESR and capacitance were set based on the measurement accuracy of the used LCR meter. If the measurement error interval of pre-immersion and post-immersion overlaps, the device passed the test. If the deviation from the reference value was lower than 5% of the reference value, the device was still monitored and deemed as a “partial failure”. When the deviation exceeded the 5% threshold, the monitoring of the unit stopped as the encapsulation failed completely. As for the diode circuit, during forward bias operation, the voltage across the diode shall be within normal values for a silicon diode at 1 mA current (around 600 mV^37^. During reverse bias testing, the threshold was set to 1 µA at 30 V and 10 µA at 30 V across the diode terminals for partial and complete failure, respectively.

### Chloroform presence testing

To verify that the chosen P3HT coating method does not introduce toxic chloroform to the device, Raman and FTIR analysis was performed. The used samples were identical to these used for biocompatibility testing (“Biocompatibility testing”).

First, using a confocal Raman microscopy at 785 nm excitation, 24 mW power and 50 × lens, a Z-scan was performed. The step was 1 μm and the maximum scan depth was 20 μm. A pure sample of chloroform and P3HT were also scanned as references for comparison. The measurement results were then plotted and analyzed statistically using multivariate statistical analysis to indicate a presence or absence of chloroform in the coated epoxy sample.

**Figure 5:**
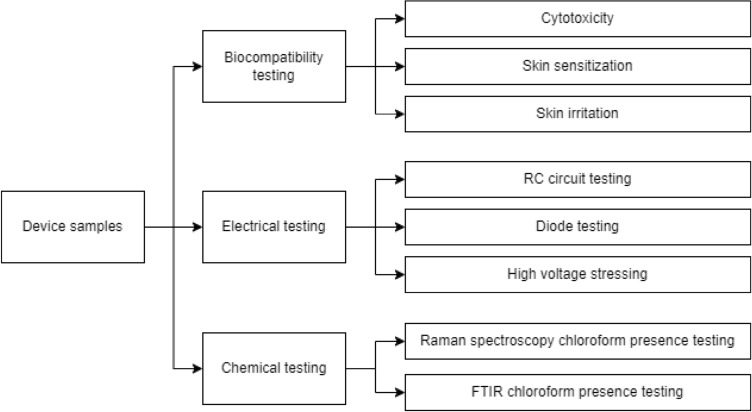
Test matrix.

Second, FTIR microscopy measurement using single reflection ATR crystal. Spectra of chloroform, P3HT and sample were then plotted and compared to provide as a supplementary source of data for determining if chloroform is present in the sample. The overall test matrix which includes biocompatibility testing, electrical testing and chemical testing is shown in Fig. 5.

## Results

The performance testing results are shown in Table 1. A complete table with measurements is provided as Supplementary Table S3. The PDMS group of devices (“PDMS1-3”) showed the lowest endurance with two devices failing at the 10-min timestamp and the other device failing at 1 h. The longer-lasting device showed signs of partial failure at the 10-min timestamp, as well.

**Table 1:** Testing results (*PF* partial failure, *CF* complete failure).

	Result	PF timestamp	PF type	CF timestamp	CF type
PDMS1	Failed	–	–-	10 min	Diode circuit, RC circuit
PDMS2	Failed	10 min	Diode circuit (1.8 µA)	1 h	Diode circuit, RC circuit
PDMS3	Failed	–	–-	10 min	Diode circuit, RC circuit
EPOXY1	Failed	–	–-	1 h	Diode circuit
EPOXY2	Failed	–	–	10 min	Diode circuit, RC circuit
EPOXY3	Failed	–	–	1 day	Diode circuit, RC circuit
HERM1	Did not fail	–	–	–	–
HERM2	Partial failure	1 day, 15 days	RC circuit (≤ 3% deviation)	–	–
HERM3	Did not fail	–	–	–	–
CTRL1	Did not fail	–	–	–	–
CTRL2	Did not fail	–	–	–	–
CTRL3	Did not fail	–	–	–	–

The epoxy-only group (“EPOXY1-3”) was the least consistent one, with the devices failing after 10 min, 1 h, and 1 day, respectively. No partial failures were observed before the complete failure of the encapsulation.

None of the devices with epoxy and hermetic feedthrough (“HERM1-3”) failed completely, all devices survived the 15-day testing. One of the devices exhibited a partial failure when after 4 h and 15 days the value of capacitance/resistance deviated around 2%/2% and 3%/1%, respectively, which exceeded the measurement error of the instrument. Subsequently, in the epoxy encapsulated devices, the diode reverse current when 100 V was applied did not exceed 1 µA, the measured values were 0.0 µA, 0.2 µA and 0.0 µA.

Finally, none of the devices in the control group exhibited partial or complete failure.

**Figure 6:**
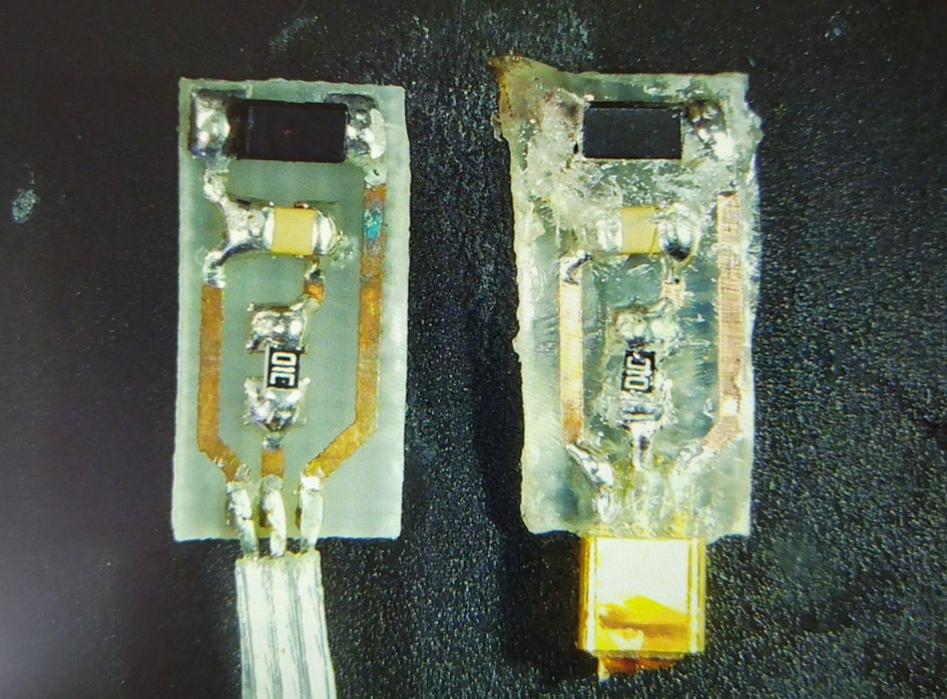
Corrosion comparison (PDMS on the left, epoxy with hermetic feedthrough on the right).

**Figure 7:**
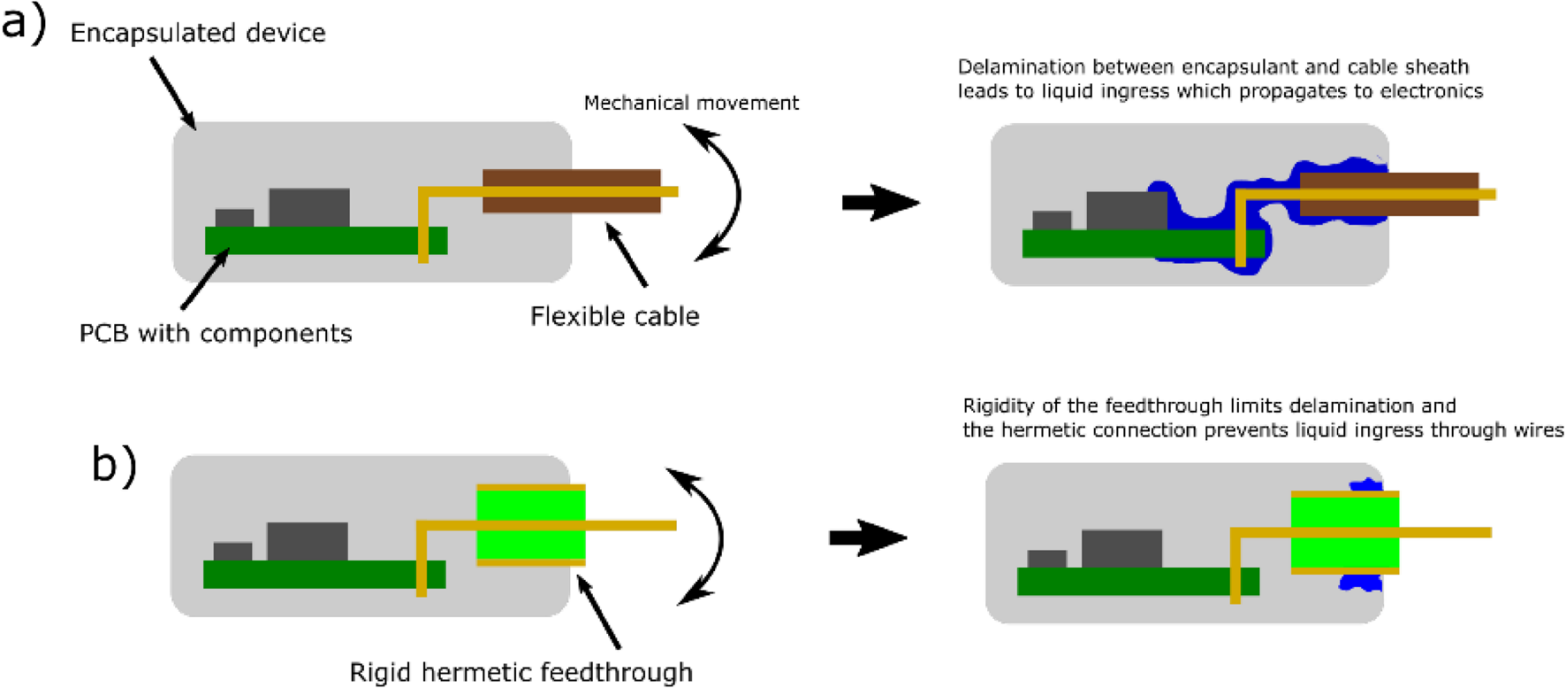
Probable water ingress path comparison—(**a**) without hermetic feedthrough; (**b**) with hermetic feedthrough.

**Figure 8:**
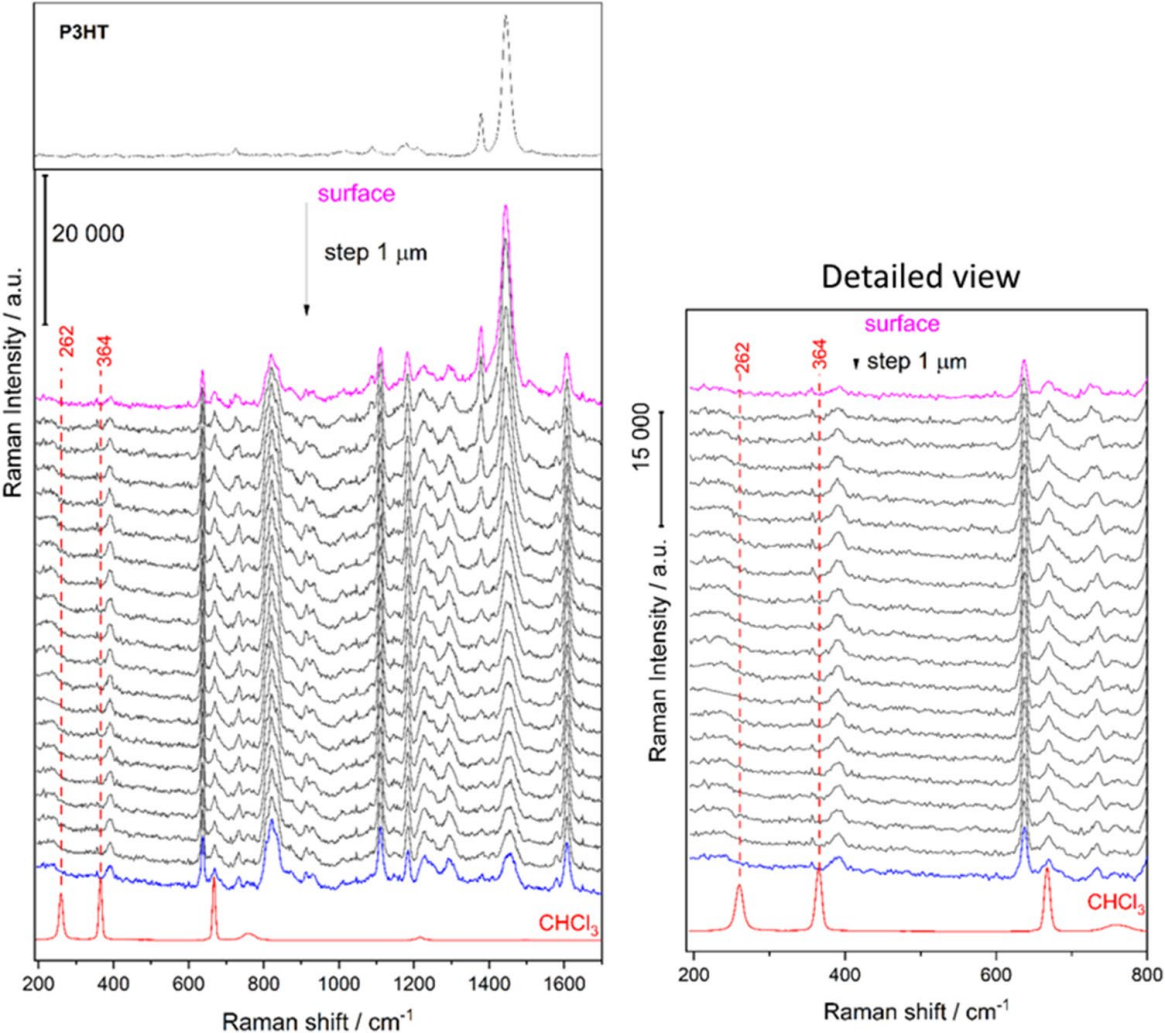
Raman spectroscopy results.

To show the visible effects of liquid ingress due to encapsulation failure, one device from the PDMS group (PDMS1) and one device from the hermetic feedthrough group (HERM1) had their encapsulant removed. While the PDMS encapsulated device could be easily freed from the silicone material with one incision, the epoxy had to be manually scraped with the use of a hot air gun at 200 °C. Even so, it was not possible to fully clean the epoxy off the circuit board without using even more heat or force. The use of force resulted in mechanical breakage of the gold-plated pins during handling, after which the removal was stopped to avoid further damage. This indicates a very strong bond of the epoxy both to the circuit board and to the soldered pads and components. The comparison is shown in Fig. 6. On the PDMS encapsulated board, copper tarnishing is present. Also, in the top-right part, a presence of green residue may indicate the presence of copper(II) salt. In the epoxy encapsulated board with hermetic feedthrough, however, the copper layer remained almost pristine. The browning of the epoxy in the top left corner was caused by heating which was required to remove it. On the hermetic feedthrough, after heating, a light brown residue was discovered. It was not the P3HT polymer as it did not readily dissolve in chloroform, bromobenzene or chlorobenzene which are the three best solvents for P3HT polymer. Also, based on the Raman spectroscopy measurements (Fig. 8), the biggest concentration of the polymer is within few micrometers beneath the surface, the polymer dissolved in the chloroform does not penetrate through the device. Also, to provide more data for the decision, heating of pure epoxy to 200 °C for period of time of more than 5 s produced brown residue on its surface that was insoluble in chloroform. Thus, the most probable theory is that the residue is a decomposition product of the epoxy or an indication of water ingress (water and/or salts reacting with some of the surrounding materials). The probable water ingress path comparison is shown in Fig. 7. Based on the results, when no hermetic feedthrough is present, delamination during demolding or further operation of the device is much more likely to cause to liquid ingress than when the hermetic feedthrough is present.

The results of in vitro cytotoxicity tests confirmed the absence of cytotoxic effects elicited by the test material extracts, the viability values obtained in two independent runs with undiluted 100% extracts were higher than 70% in comparison with untreated control (implant-epoxy 76.4% and 96.3%, implant-p3ht 90.5% and 90.4%, respectively).

The results of intracutaneous (intradermal) irritation in vivo did not show the potential to produce irritation following intradermal injection of extracts of the tested materials, as the final test samples scores were lower than 1.0 (0.22 and 0.06 for implant-epoxy in saline and oil, respectively; 0.00 and 0.11 for implant-p3ht in saline and oil, respectively).

The results of skin irritation in a group of human volunteers confirmed substantially lower frequency of the skin irritation in the case of the test materials application than in the case of the positive control, i.e. the tested samples/prototypes designated as implant-epoxy and implant-p3ht were not regarded as substantial skin irritants. No skin reactions (erythema, edema, dryness) were recorded for any of the tested materials.

The *in chemico* direct peptide reactivity assay (DPRA) revealed no sensitization potential of the samples as the cysteine depletion values did not reach the cut-off value for the cysteine depletion model (i.e. 13.89%), the mean depletion value for implant-epoxy was 1.3% and for implant-p3ht 1.4%.

The in vitro skin sensitization test LuSens did not reveal any sensitization potential for the samples extracted in D-MEM or DMSO. Detailed graphs are available as Supplementary Material S4. The bars represent the value of luciferase activity induction and the dashed line represents the cut-off value of the induction of luciferase activity (1.5-fold change). The dotted lines represent the viability of the samples at the used concentrations and the solid line represents the cut-off value of the viability (70%). The luciferase activity did not exceed a 1.5-fold induction.

As for the skin sensitization in vivo—LLNA:DA—the Stimulation Index (SI) values of the tested materials were lower than 1.80, i.e. the cut-off value for prediction of sensitizing potential according to Council Regulation (EC) No. 440/2008. SI for implant-epoxy was 0.58 in saline and 1.26 in oil; SI for implant-p3ht was 1.76 in saline and 1.24 in oil. As no sample produced SI higher than 1.8, both test materials are considered to have no skin sensitization potential in vivo.

**Figure 9:**
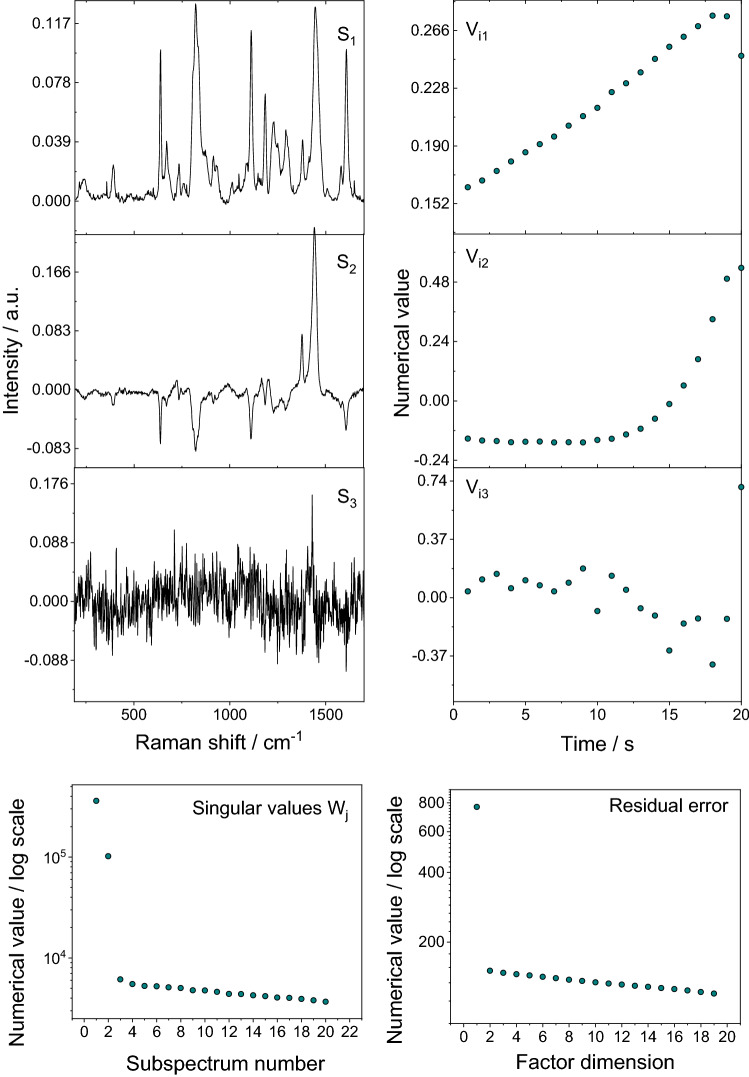
Multivariate analysis of Raman spectroscopy results.

The results of Raman spectroscopy to determine the presence of chloroform in coated epoxy samples are presented in Fig. 8. The 667 cm^−1^ band of chloroform overlaps with the epoxy, thus 262 cm^−1^ and 364 cm^−1^ bands were used for analysis. A zoomed detail of the plot is also provided to show the results more clearly. A multivariate statistical analysis (Fig. 9) was done to assess the presence of chloroform in sample. Both visual and multivariate analysis did not show presence of chloroform in the measured samples.

**Figure 10:**
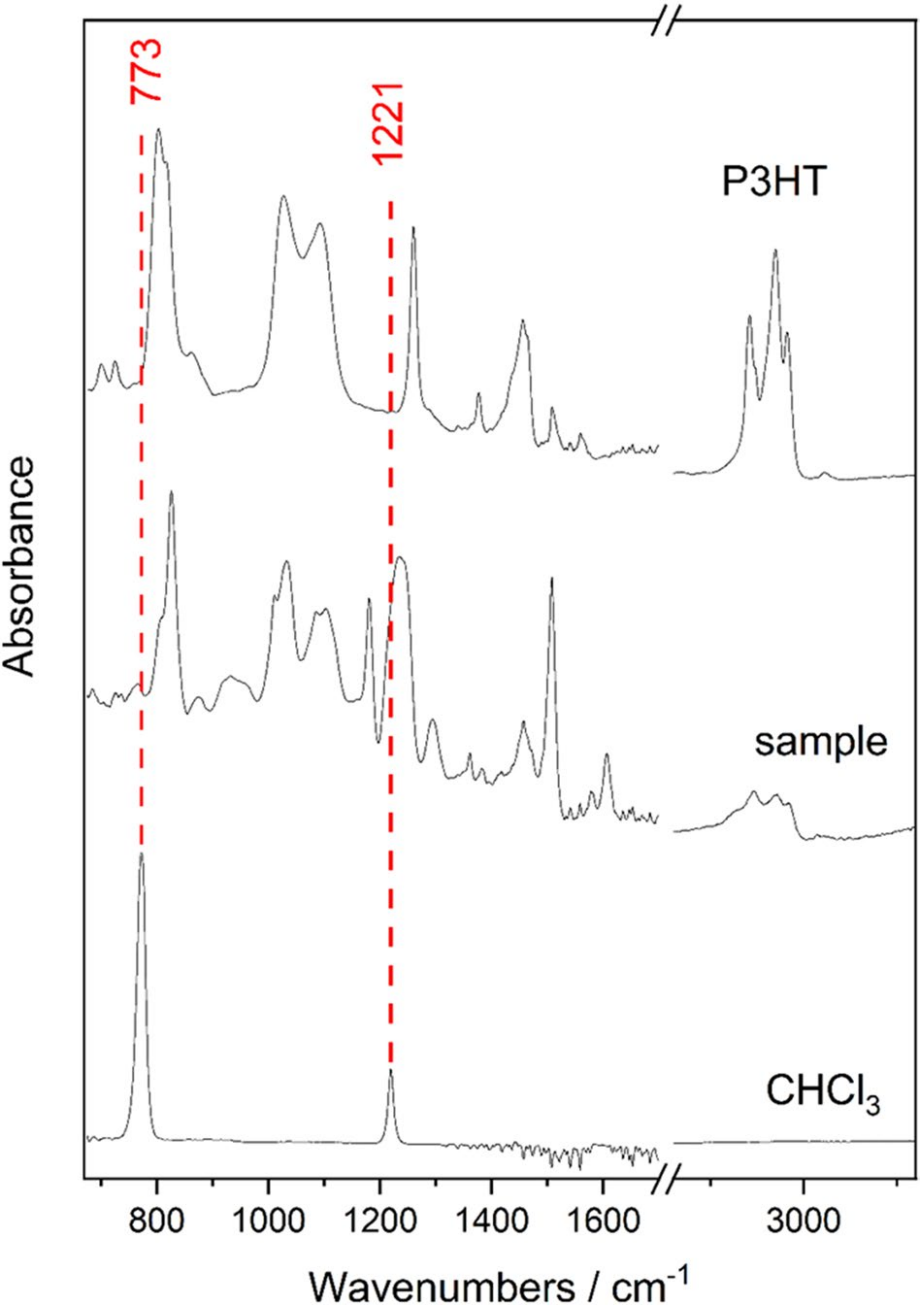
FTIR measurement.

The results of FTIR measurement are shown in Fig. 10. The 1221 cm^−1^ band overlaps with the epoxy and cannot be used for analysis. However, the 779 cm^−1^ band overlaps with P3HT minimally and does not overlap with epoxy. The results provide a supplementary proof that chloroform does embed to the P3HT polymer or the epoxy.

## Discussion

The method of encapsulation successfully passed various tests according to ISO10993. It was biologically evaluated according to ISO10993 for cytotoxicity, skin sensitization and skin irritation. Different evaluation methodologies—in accordance with the specific test requirements—in vivo, in vitro and in chemico—were used and none of them showed any signs of cytotoxicity or skin irritation/sensitization potential. This was shown both for P3HT coated and uncoated samples. According to the Annex A of ISO10993-1^38^, the spectrum of performed tests matches all recommended categories for implantable devices to tissue or bone for limited periods of time (< 24 h) for use in humans. However, before any medical device is approved for use in the EU, the final product has to be subjected to preclinical evaluation to fulfill the requirements of Regulation (EU) 2017/745 of the European Parliament and of the Council, on medical devices (MDR). Similar regulations are enforced in other parts of the world. That is, however, outside of the scope of the uses discussed in this article.

The P3HT coating provides a barrier layer formed by a polymer that is known to be biocompatible, even when present inside the cytoplasm of cells in the form of nanoparticles^18^. The main advantage of this polymer is that it can be easily functionalized to tailor the cell-surface adhesion properties^17^.

**Figure 11:**
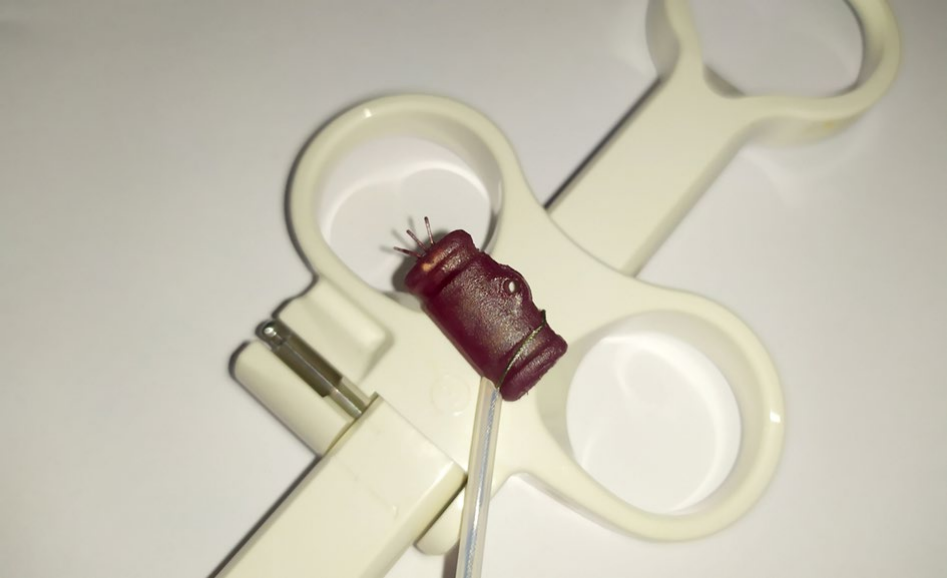
Example of a grasping feature embedded in the encapsulated device.

It was demonstrated that a very small electronic package can be successfully encapsulated with wall thickness as low as 0.4 mm which puts this method on par with simple heat shrink tubing encapsulation while achieving biocompatibility and freedom of shape. The fact that the shape of the encapsulated device can be easily modified by milling additional features to the mold can be extremely useful both for endoscopic and laparoscopic implantations. Recesses or even holes for graspers and snares can be easily implemented with this method, as seen in Fig. 11. Unlike current methods which resort to brushing/spraying or simple potting using a one-piece mold, a fully non-traumatizing shape can be created, reducing risk of rejection due to mechanical damage of tissue.

The performance measurements clearly showed that for small devices with small wall thicknesses, the presented method is superior to simple PDMS or epoxy potting without hermetic feedthroughs. According to expectations, PDMS potting did not provide sufficient protection and failed first. The epoxy-only encapsulation, on average, did last longer but the method was not consistent. This may be explained by the fact that the removal of the encapsulated electronics from the mold does require a small force which directly translates to the bond between the cable and epoxy. Cracks, delamination, or similar failure modes can occur in this area. The variant with hermetic feedthrough was shown to be superior, with one device showing signs of partial encapsulation failure. However, the change of circuit parameters was in a lower single-digit percentage.

An interesting fact was that almost all devices that failed did so suddenly, not gradually. This can be explained by the fact that the circuit failure was connected directly to delamination between the cable and epoxy/PDMS and subsequent capillary ingress of the liquid to the surface of the PCB, not transmission of water vapor through the material with gradual condensation on the PCB.

Contrary to common belief, the use of a toxic solvent (chloroform) for P3HT coating did not impair the biocompatibility of the device at all. The performed in vitro and in vivo tests confirmed excellent biocompatibility of both samples. This can be explained by the fact that due to very high volatility, the chloroform fully evaporates during the heating step and does not embed into the structure of the P3HT polymer or the epoxy. The total coating time until the solvent visually evaporates is less than 15 s which also limits the possible diffusion of the solvent to the epoxy. This was confirmed by Raman spectroscopy and FTIR measurement which showed no residual chloroform in the coated samples. The wet-coating methods incurs some environmental concerns. However, the fact that this method is intended for prototypes, should outweigh the disadvantages due to its simplicity. Next, the P3HT coating is not mandatory, it is primarily used to create a surface that is known to be tailorable according to final applications^18^. The epoxy itself applied by the described method is biocompatible on its own. This method is also economical. Apart from the 3-axis CNC mill, it does not require any special equipment. The total material cost for the encapsulation of one presented device was around 21 USD. This includes the epoxy cost of 0.81 USD (65 USD for the 50 ml epoxy cartridge, roughly 1.25 ml of epoxy was used for two devices) and 0.02 USD for the P3HT polymer (120 USD per gram of P3HT, roughly 0.1 ml of the ink was used for two devices which translates to 0.2 mg of the polymer). The cost of the hermetic feedthrough was 20 USD. The cost of the material for the mold fabrication was 6.32 USD (the cost of a 1 m long PVDF rod was 155 USD, around 4 cm was used for mold fabrication; the cost of a 1 m PTFE rod for side pin milling was 2 USD, 6 cm was used). The wear of the end mills and ball mills used for fabrication was negligible. From the equipment point of view, according to our experience, a small import 3-axis CNC mill can be obtained for less than 1000 USD, including economical HSS (high-speed steel) tools and a small machining vise.

It should be noted that the electronics used to test the encapsulation method was intentionally made as vulnerable as possible to the environment. This was done to limit the number of factors that could distort the measured data. The durability of the electronics can be further enhanced by conventional means, i.e. providing solder mask to protect the thin copper tracks, plating of exposed copper with inert coatings (most commonly electroless nickel immersion gold plating—ENIG) and/or spraying the populated PCBs with a layer of conformal coating. Using these techniques, the lifetime can be expected to be increased. The used epoxy is also resistant to many organic and inorganic solvents and was also tested for resistance to high-temperature treatment (heat aging) at temperatures of up to 177 °C for extended periods of time without losing bond strength to metal^39^. Thus, soldering to the hermetic feedthrough will not negatively affect the epoxy bond to it. As for dielectric strength, the encapsulated structure was tested up to 333 V/m which should satisfy requirements of vast majority of small implantable devices, including wireless communication where the radiated RF power is logically limited. The encapsulant—Loctite EA M-31 CL—was tested by manufacturer for dielectric breakdown voltage with measured value of 19.7 kV/mm^39^ that is almost an order of magnitude larger than that of air.

The presented method is easily adaptable to various types and shapes of implantable devices, including complex ones. After careful consideration of the material type and repeated biocompatibility testing, the mold parts can be also manufactured by 3D printing. However, CNC machining usually results in higher accuracy of the parts and overall consistency of the process. The hermetic feedthroughs can be obtained in different sizes and types, enabling the realization of complex multi-channel sensor or neurostimulation systems. Researchers using this method are also encouraged to experiment with various epoxy resins. The epoxy which was used in this paper is widely available in small economical cartridges directly compatible with static mixers. Other types of biocompatible epoxies that can be used include Epotek Med-301-2, Master Bond EP30-4Med, Opti-tec 5006-1 and others. The epoxy should be chosen regarding the target application of the device. Next steps that can lead to successful deployment to mass manufacturing practice can include addition of ejection system (like conventional plastic injection molds) to simplify the demolding process. A mold machined from metal (preferably steel) can provide extended life but can be prone to epoxy bonding to it. Other biocompatible polymers can be applied using the wet-coating method to tailor the properties of the surface of the resulting device.

## Conclusion

The findings showed that the presented encapsulation method offers a better alternative to the commonly used PDMS or epoxy potting methods without the use of custom machined mold. The CNC machined mold allows a complete freedom of shape and the addition of a hermetic feedthrough considerably improves endurance against liquid ingress due to delamination and/or insufficient bond between the encapsulant and wires. Moreover, the method was qualified according to ISO10993 (ISO 10993-5, ISO 10993-10, ISO 10993-12 and ISO 10993-23), a relevant standard for biological evaluation of implantable medical devices. Overall, this method was proven to be quick, economical, effective, and ensures biocompatibility of the encapsulated devices. Thus, it provides researchers a valuable tool to quickly create prototypes of novel active implantable devices that are safe for pre-clinical testing and limit implant rejection (both from biocompatibility point of view and non-traumatizing shape of the device). Future work and expansion of this method may focus on development of encapsulants with enhanced adhesion to noble metals or developing additional protection measures that can be implemented to the printed circuit board itself, such as conformal coating.

## Supplementary Information

Supplementary Information 1.

Supplementary Information 2.

Supplementary Information 3.

## Data Availability

All data needed to reproduce the method is provided in the article or as a supplementary material.
