# Supplementary material for: Low-cost and prototype-friendly method for biocompatible encapsulation of implantable electronics with epoxy overmolding, hermetic feedthroughs and P3HT coating
Source: Sci Rep. 2023 Jan 30;13:1644. doi: 10.1038/s41598-023-28699-6 (PMC9887057; doi:10.1038/s41598-023-28699-6)

**Low-cost and prototype-friendly method for biocompatible encapsulation of implantable electronics with epoxy overmolding, hermetic feedthroughs and P3HT coating**

Supplementary figure S1: Schematic diagram and printed circuit board layout


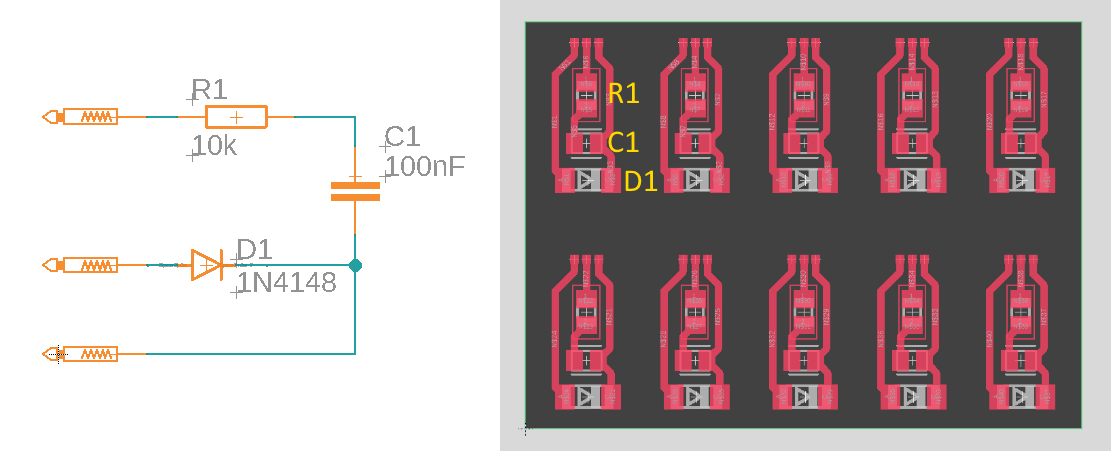


**Low-cost and prototype-friendly method for biocompatible encapsulation of implantable electronics with epoxy overmolding, hermetic feedthroughs and P3HT coating**

Supplementary information S2: STEP 3D model of the mold

*Provided separately as S2.step file*

**Low-cost and prototype-friendly method for biocompatible encapsulation of implantable electronics with epoxy overmolding, hermetic feedthroughs and P3HT coating**

Supplementary table S3: Table with measurements from performance testing

*Provided separately as S3.xlsx file*

**Low-cost and prototype-friendly method for biocompatible encapsulation of implantable electronics with epoxy overmolding, hermetic feedthroughs and P3HT coating**

*Supplementary figure S4: Luciferase activity and cell viability of the tested extracts a) in D-MEM, b) in DMSO.*


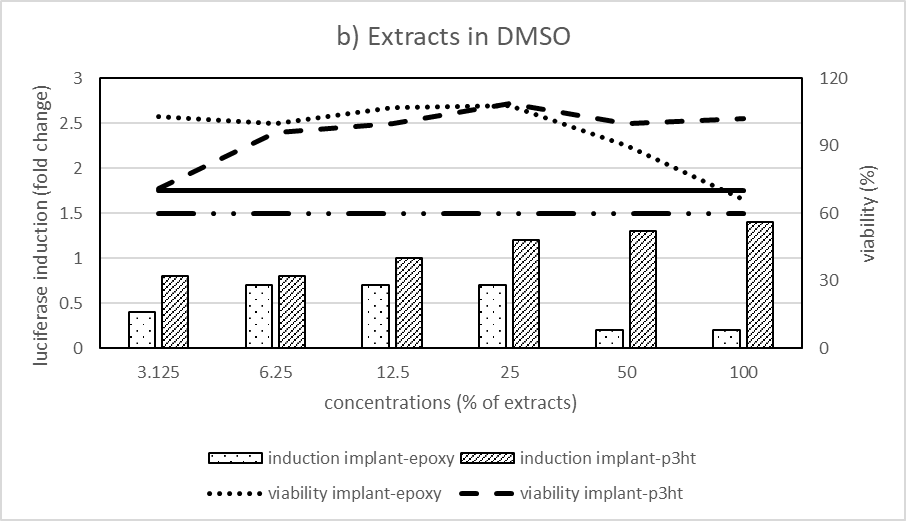

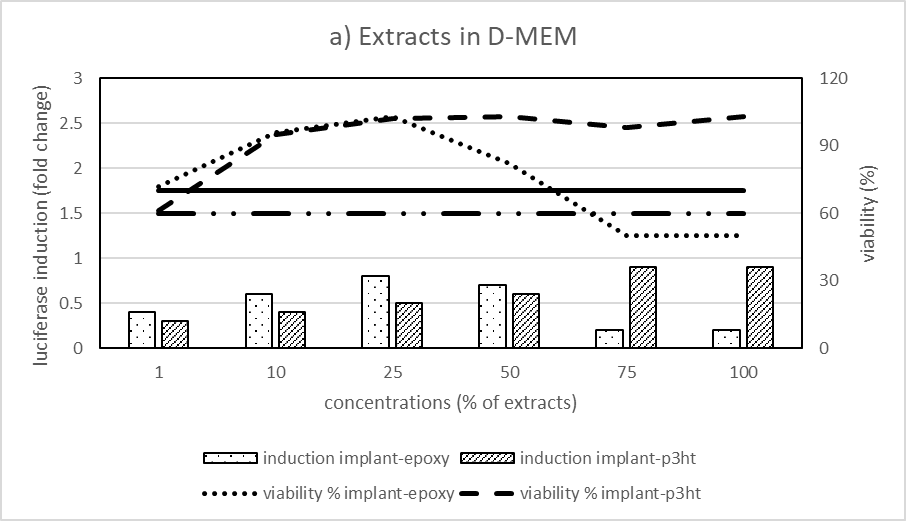

Supplement: Supplementary file 1 — Supplementary Information 1. [file 41598_2023_28699_MOESM1_ESM.docx]
